# A Review of Historical and Future Changes of Extratropical Cyclones and Associated Impacts Along the US East Coast

**DOI:** 10.1007/s40641-015-0013-7

**Published:** 2015-07-01

**Authors:** Brian A. Colle, James F. Booth, Edmund K. M. Chang

**Affiliations:** 1 School of Marine and Atmospheric Sciences, Stony Brook University/SUNY, Stony Brook, NY 11794-5000 USA; 2 Department of Earth and Atmospheric Science, City University of New York (CUNY), City College, USA

**Keywords:** Extratropical cyclones, Storm surge, US East Coast, Climate Change

## Abstract

This paper reviews the historical and potential future trends of extratropical cyclones (ECs) along the United States (US) East Coast and western Atlantic, as well as potential changes in coastal flooding, heavy precipitation, and damaging winds. Most models project a steady decrease in the number of ECs for the US East Coast and western Atlantic region by the middle to later twenty-first century, while there is an increase in more intense (<980 hPa) cyclones and heavy precipitation; however, there is also been large interdecadal and interannual variability. Potential biases may exist in the models because of difficulty capturing: (a) the Atlantic storm track sensitivity to the Gulf Stream SST gradient, (b) latent heating within these storms, and (c) dynamical interactions at jet level. More work is needed to determine future changes in hybrid storms (e.g., Sandy 2012) and diagnostics to better understand the future cyclone changes in the models.

## Introduction

### Background

Extratropical storm tracks and their associated cyclones and fronts are responsible for much of the high-impact weather over the mid-latitudes. Several studies have shown that the frequency of extreme precipitation, high wind, extreme cold events, and coastal flooding are strongly related to variations in the extratropical storm track activity, especially during the cool season (but also to a lesser extent during the warm season). For example, on the global scale, Pfahl and Wernli [1] showed that over most extratropical regions, precipitation extremes (defined as the top 1 % of daily precipitation) are frequently coincident with occurrences of cyclones, with “hot spots” over the storm track entrance regions close to the Northeast USA and Japan. Over the USA, Kunkel et al. [2] subjectively categorized the meteorological cause for extreme precipitation events (defined as 1-in-5-years events) over the contiguous USA for the period 1908–2009 by examining long-term gauge data at 935 stations and daily weather analyses and concluded that 78 % of these events are caused by extratropical cyclones either near the low center or near a front.

Given its location relative to the common paths of extratropical cyclones, the US East Coast is especially vulnerable, particularly for “nor’easters,” which are storms that develop as they move north near the coastline. The December 1992 extratropical cyclone [3, 4] illustrated the damage coastal flooding can incur along coastal southern New England [3]. More recently in late October of 2012, Hurricane Sandy underwent an extratropical transition and made landfall on the New Jersey shoreline on the 29th of October 2012 [5]. Sandy’s landfall produced a catastrophic storm surge stretching from New Jersey to Rhode Island that made it the sixth costliest US tropical cyclone on record since 1900 [6]. In spite of the incredible damage Sandy generated, two recent modeling studies demonstrated that Sandy’s storm surge was not the worst-case scenario for the NY/NJ Bight [7, 8].

There has been extensive research on the climatology and physical processes associated with these US East Coast and western Atlantic winter storms. Mather et al. [9] conducted one of the first temporal climatologies of eastern US cyclones using coastal storm reports, weather summaries, and water damage reports contained in periodicals and newspapers. They estimated a moderate to major storm to impact New York and New Jersey to occur on average every 1.4 years. Reitan [10] illustrated the broad climatological maximum of mid-winter cyclogenesis off the southeast US coast along the Gulf Stream. Colucci [11] and Zishka and Smith [12] showed the maximum in cyclogenesis parallel to the coast about 5° offshore from South Carolina to the Canadian Maritime Provinces. Davis et al. [13] used wave heights greater than 1.6 m along the East Coast to obtain the synoptic climatology of winter storms given its connection to beach erosion. Field experiments were conducted to better understand frontal structure and role of latent heating, such as the Genesis of Atlantic Lows Experiment (GALE) [14] in 1986 and the Experiment on Rapidly Intensifying Cyclones over the Atlantic (ERICA) [15] in 1989. Much of the severe weather occurs on the mesoscale (<500 km), such as heavy snowbands [16], flooding rains [17], and storm surge [4]. Coastal areas of the Northeast USA are extremely vulnerable to storm surge, with the problem likely to become worse as sea level rises during the next 100 years [18].

Cyclones affecting the Northeast USA generally propagate along three distinct tracks [10, 12]: Alberta Clippers that form over western Canada and track eastward across southern Canada, Colorado Lows that form over the US southwest and tracks northeastward towards the Great Lakes, and nor’easters that form either over the gulf coast or off the Carolinas that track north-northeastward along the coast. Milrad et al. [19] suggested that along the east coast, extreme precipitation events are associated with nor’easters, while Sisson and Gyakum [20] suggested that over inland areas, heavy precipitation events are more related with cyclones from the southwest. Extreme wind events over the northeast are generally associated with storms from the southwest with the cyclone center to the west of the affected regions [21–23], while Colle et al. [18] suggested that moderate storm surge events at New York City are mainly caused by nor’easters.

There has been growing interest and research on regional climate change over the US East Coast because extreme temperature, wind, and coastal flooding impact numerous human, industrial, commercial, and marine ecosystems. Given the prevalence of storms in the regions and the strong connection between the extratropical cyclones and extreme weather, these storms have been evaluated in climate models. There have also been several studies investigating US East Coast and western Atlantic cyclone trends in model projections, but there has not been a review of these results and future directions for research.

### Motivation

Mid-latitude storm tracks are an important part of the global circulation [24]. Synoptic eddies transport large amounts of energy poleward, which affects the climate on seasonal and decadal timescales if there are changes in the storm tracks. There are complex changes in future mid-latitude storm tracks for increased levels of carbon dioxide (CO_2_) [25]. Baroclinic instability is impacted by the horizontal temperature gradients and static stability. Low-level temperature gradients are expected to weaken (except Northern Atlantic) in a warmer climate due to high-latitude warming, whereas the temperature gradients in the upper troposphere are expected to strengthen [26]. Thus, it is unclear which of these two opposing changes will dominate future changes in storm activity [27, 28]. Meanwhile, previous studies have shown strong influence of moist processes on mid-latitude storm dynamics, with increasing latent heat release in the warm sector of a storm providing an additional source of available potential energy and stronger storms [25, 29].

Many of the studies on future changes in mid-latitude storms have utilized global climate models (GCMs). There are some pros and cons with using global models to determine future extratropical cyclone changes that need to be discussed in light of recent studies. Also, as computer power increases, there will be more dynamical downscaling of climate model predictions with mesoscale models or higher resolution GCMs. There are already some attempts to do this, which is highlighted below, but there are some challenges and opportunities.

There have been some recent review articles on extratropical cyclones over the North Atlantic [30, 31]. Feser et al. [30] showed that there is an increase in storm numbers during the last 4–6 decades, but there is large decadal variability for the last 100–150 years. Most of the increase in cyclones in recent decades has been north of about 55–60° N, while there are decreases south of 50° N. Therefore, the goal of this paper is not to review all extratropical cyclone literature over the Atlantic but rather focus on cyclones impacting the US East Coast and how they may change in the future, since this is a densely populated region with potentially large impacts. This review article will address the following questions:

- What is the skill of GCMs in predicting cyclones and precipitation over this US East Coast and western Atlantic regions, and how does the model variance impact some of the future predictions?
- What are some of the issues limiting the skill of GCMs for these storms and impacts?
- What is the role of latent heating in the frequency and intensity of future storm projections, and how might a dynamical downscaling effort help?
- What are some potential impacts from these storms, such as heavy precipitation and coastal flooding?
- What are the future research directions for East Coast winter storms during climate change?

Section 2 will present some of the datasets, as well as historical and future changes in East Coast and western Atlantic cyclones. Section 3 will present some challenges of predicting these storms using GCMs given resolution issues and uncertainties from physical processes, such as latent heating. It will also discuss attempts to address these issues using dynamical downscaling. Section 4 will discuss some of the future precipitation and coastal flooding changes given the models. Section 5 will summarize and offer some future directions for this field.

## Historical and Future Changes

### Data and Methods

Evaluating changes in extratropical storms over several decades requires an automated procedure to identify these cyclones. Some have defined storm tracks using a Lagrangian approach by tracking individual cyclones in a reanalysis or model gridded dataset manually [32] or automated approaches [33, 34]. There are numerous uncertainties associated with the cyclone tracking, and attempts have been made to intercompare different algorithms [35]. There tends to be less agreement for relatively weak cyclones and more agreement with deep cyclones. As noted by Eichler and Gottsschalck [36], there is also sensitivity to the reanalysis dataset used. For example, the ERA-INTERIM with its higher resolution produced a greater number of smaller (<200 km) cyclones than the ERA-40 and NCEP/NCAR reanalysis data.

Another approach is more Eulerian, since it uses a band pass filter as introduced by Blackmon [37] to highlight time scales of 2 to 6 days of the variance of different eddy quantities, such as meridional wind and eddy kinetic energy [24, 38]. Unlike the tracking method, this technique captures all storm activity; however, both the low and the high-pressure systems in the vicinity of the storm track are included with this method [39]. Given these uncertainties, some studies use both Eulerian eddy statistics and Lagrangian cyclone tracking [40, 41].

Data from phase 5 of the Coupled Model Intercomparison Project (CMIP5) [42] are available at 6-h intervals, and for most models, high vertical resolution data on the model’s own grid are available; these allow for the tracking of cyclones and the computation of process budgets. There is a historical period from the mid-nineteenth century to 2005 forced by observed atmospheric composition changes (reflecting both anthropogenic and natural sources) and time-evolving land cover. Each of the models is also run for different emission scenarios or representative concentration pathways (RCPs) for the future to 2100 or later. The CMIP5 models have been analyzed for several different phenomena, such as temperature, precipitation, storm tracks, droughts, floods, etc., around North America for both the historical [43] and the future periods [44].

### Historical Trends

During the past 50 years, there has been a large interdecadal variability in extratropical cyclones (ECs) and storm surge along the East Coast [13, 18, 45, 46]. Using wave heights as a proxy, Davis et al. [13] found that the frequency of nor’easters declined from the 1950s to a minimum in the 1970s, followed by a subsequent increase in the 1980s. After a relatively active period in the 1990s for storm surge (4–5 flooding events around NYC), there were no significant coastal flooding events from 1997 to 2009 [18]. The North Atlantic Oscillation (NAO) has a strong relationship with the mean winter climate of the East Coast of North America [47]. During the positive phase of the NAO, there is enhanced westerly flow over the North Atlantic and a northward shift of the mid-latitude storm track [48].

El Nino Southern Oscillation (ENSO) can modify flow patterns across the hemisphere, including change in storms and storm tracks. Hirsch et al. [46] completed a ~50-year climatology of East Coast winter storms using the NCEP global reanalysis and found inter-annual variations associated with ENSO (El Nino favors more nor’easters) as well as relatively large interdecadal variations. DeGaetano et al. [48] found that East Coast storms are more frequent when El Nino is in the positive phase. Eichler and Gottschalck [36] also found that El Nino affects cyclone frequency, with more cyclone tracks during a positive ENSO, especially along the southeast US coast and Gulf Stream.

**Fig. 1:**
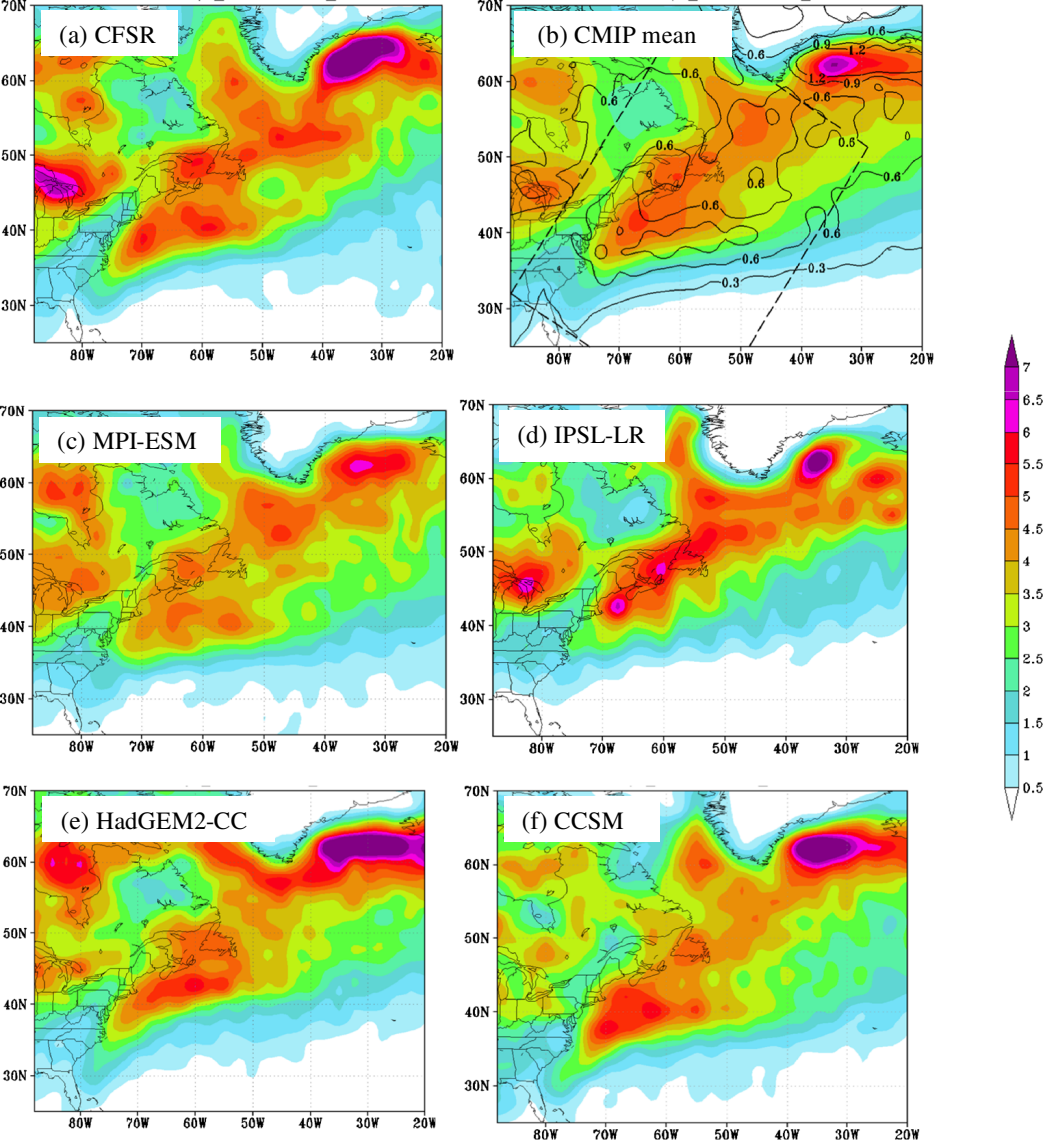
**a** Cyclone density for the CFSR analysis showing the number of cyclones per cool season (November to March) per 50,000 km^2^ for 1979–2004. **b** Same as (*a*) except for the mean (*shaded*) and spread (*contoured every 0.3*) of 15 CMIP5 models listed in Colle et al. (2012). Same as (*a*) except for the **c** MPI-ESM, **d** IPSL-LR, **e** HadGEM2-CC, and **f** CCSM models

**Fig. 2:**
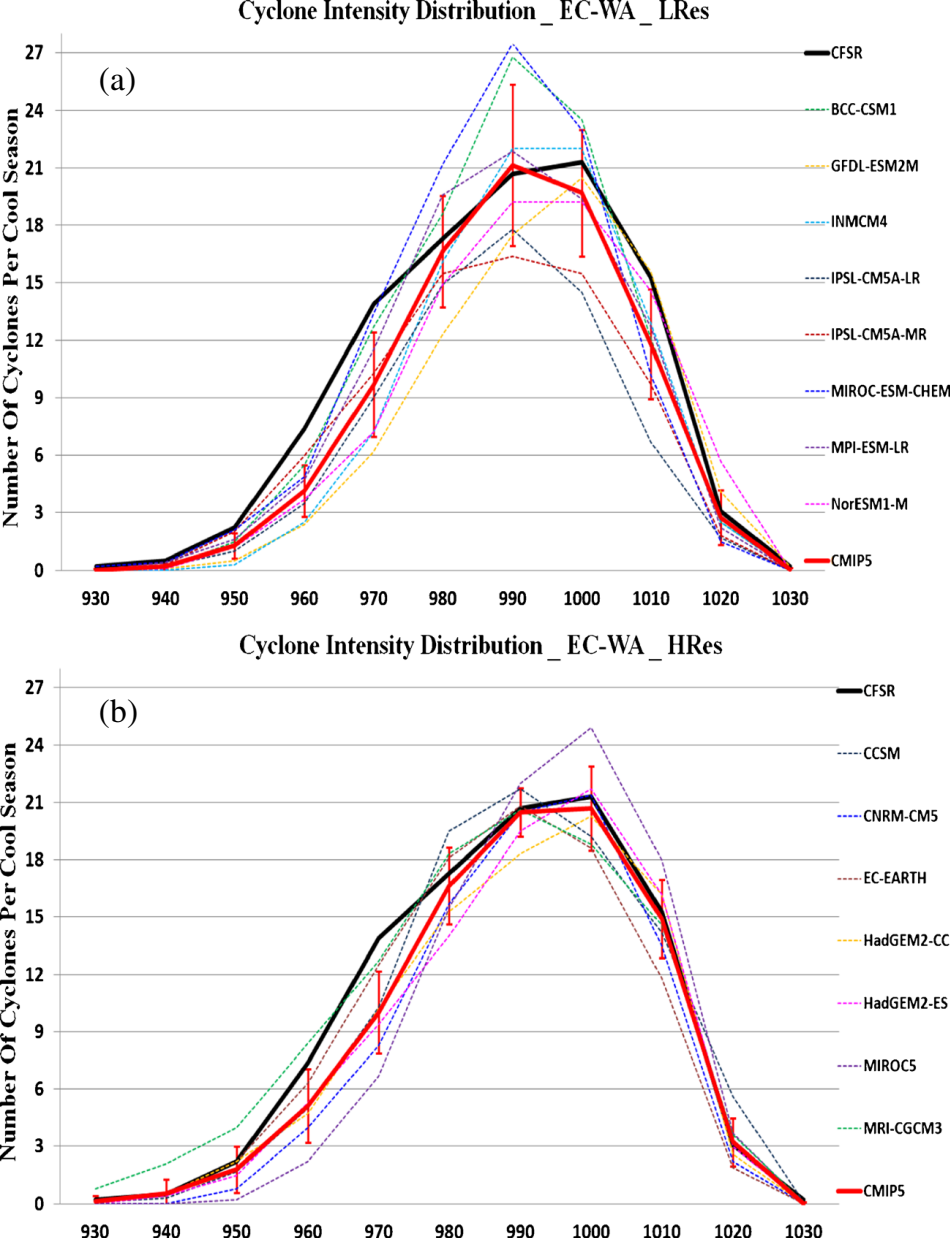
**a** Number of cyclone minimum central pressures for the 1979–2004 cool seasons within the EC-WA box region in Fig. 1 for a 10-hPa range centered every 10 hPa showing the CFSR (*bold black*), CMIP mean of the low resolution models, and each of the low resolution models. **b** Same as (*a*) except for the higher resolution CMIP5 models. Obtained from Colle et al. [34]. “© American Meteorological Society. Reprinted with permission”

Colle et al. [34] recently investigated the extratropical cyclone track density, genesis frequency, deepening rate, and maximum intensity distributions over the Eastern North America and the western North Atlantic for 15 Coupled Model Intercomparison Project phase 5 (CMIP5) models during the historical period (1979–2004). The cyclones were identified using an automated tracking algorithm applied to sea level pressure every 6 h. It was found that 6 of the top 7 CMIP5 models in terms of matching cyclone statistics with reanalysis were the models with the highest spatial resolution. Figure 1 shows the 1979–2004 cyclone track density during the cool season for the CFSR, CMIP5 mean, and select CMIP5 models from Colle et al. [34] and Sheffield et al. [43]. It highlights that the CMIP5 mean is able to get the general storm track density, but there is a relatively large variance between models. The finer resolution models (CCSM and HadGEM) had a more realistic cyclone density than the coarser resolution models (MRI-ESM and NorESM). The finer resolution models also had a more realistic distribution of cyclone intensity as determined using the storm central pressure (Fig. 2) and more realistic cyclogenesis and deepening rates [34]. However, all models underpredicted the number of relatively deep cyclones (<980 hPa). For the historical period, there was no trend in the number of US East Coast and western Atlantic cyclones (not shown).

### Future Predictions

GCM projections have been analyzed to estimate how the number and intensity of extratropical cyclones might change in the future. However, as shown by Colle et al. [34] and other studies, there is large uncertainty in these models and their twenty-first century projections. The uncertainty results not only from how these models predict the storm tracks but also how well future behavior of ENSO and other low-frequency variabilities are simulated. Therefore, although this section summarizes the future changes in these cyclones using the latest state-of-the-art models, the caveat is that the future changes may not be in the ensemble envelope given these uncertainties. Many studies project a decrease in the number of extratropical cyclones globally and over the Northern Hemisphere with global warming [49–54]. However, the future intensity of these storms is less certain, with some studies suggesting increasing intensity [49, 50, 53], while others project decreasing intensity [52].

**Fig. 3:**
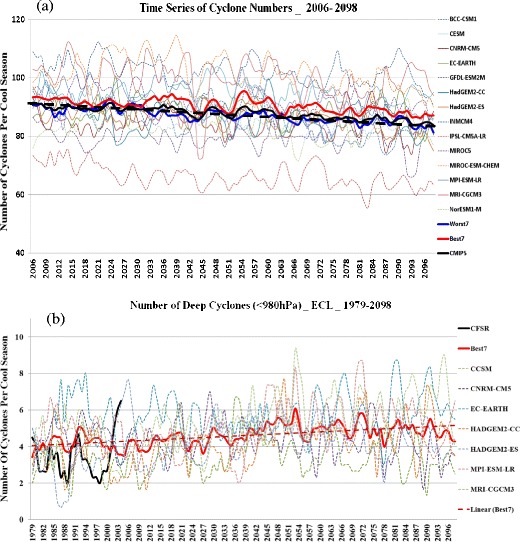
**a** Time series for the 2006–2098 cool seasons showing the numbers of cyclones per year within the EC-WA box in Fig. 1 for the mean of all CMIP5 members, Best7, Worst7, and individual members (Best7 members *solid* and Worst7 *dashed*). A linear fit (*dashed*) is made for the CMIP5 mean (*black*). **b** Same as (*a*) except for the number of relatively deep cyclones (<980 hPa) for Best7 models and mean in the EC-L region for the 1979–2098 period. Obtained from Colle et al. [34]. “© American Meteorological Society. Reprinted with permission”

Most studies have shown a decrease in the number of mid-latitude cyclones around North America [34, 55–58]. For example, Colle et al. [34] highlighted this steady decrease for the US East Coast and western Atlantic region (Fig. 3a). The reduction of the number of cyclones within the storm track is attributed to the polar amplification of warming in the lower troposphere, which in turn reduces the horizontal temperature gradient [59–62]. Meanwhile, in the subtropics, there has been an increased upper tropospheric warming and increased stability, which also favors less cyclone activity. Other analyses by Mickley et al. [63] and Leibensperger et al. [64] point to reduced frequency of summer extratropical cyclones as well as displacements of tracks towards the north. These potential cyclone changes need to be investigated using downscaled higher resolution climate models in an ensemble framework and focused more on more regional scales.

**Fig. 4:**
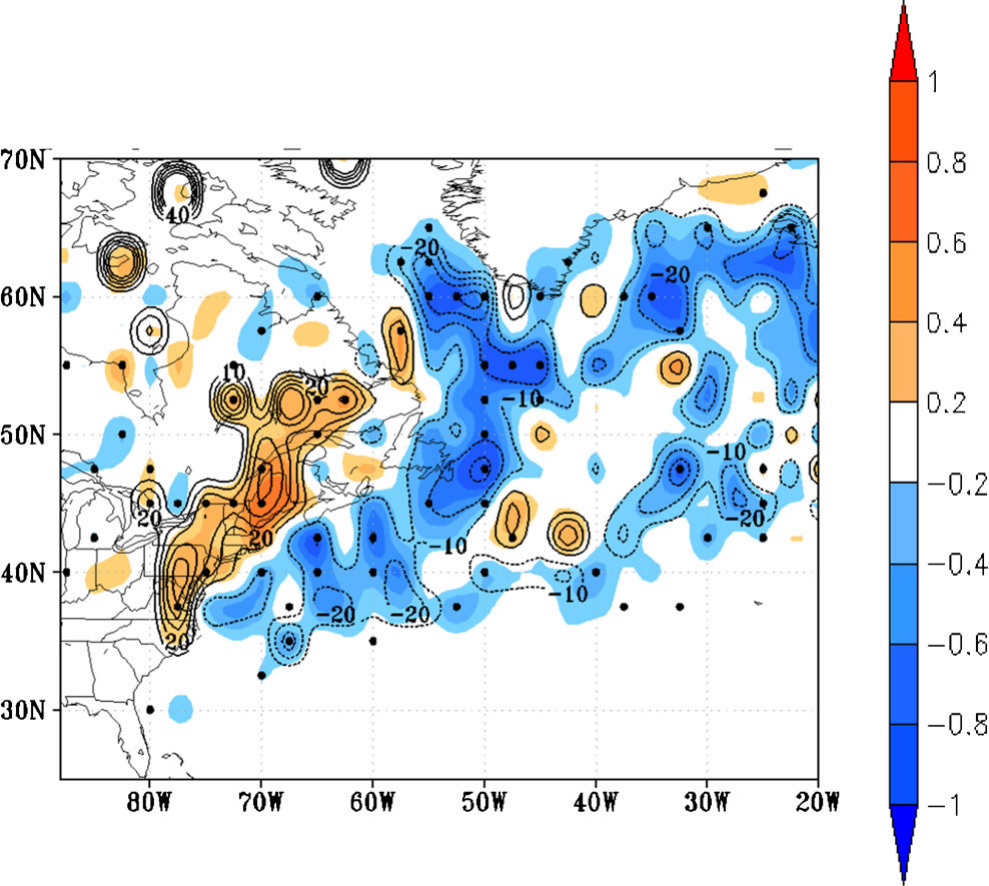
Change in the number of 6-h cyclone deepening rates >5 hPa (shaded as the number of cyclone tracks per 5 cool seasons) per 50,000 km^2^ and the percentage change (contour every 10 % with *negative dashed*) between the 2039–2068 future period minus the 1979–2004 historical period. The dots are locations in which 6 of the 7 Best7 models agree with the sign of the change. Obtained from Colle et al. [34]. “© American Meteorological Society. Reprinted with permission”

An increase in intense cyclones is limited to some areas, depending on models and experiments. For example, for predictions into the later twenty-first century, Chang [58] found that the frequency of strong cyclones projected to decrease by 15.9, 6.6, 32.6, and 16.9 % for winter, spring, summer, and fall, respectively, over North America. Regions near the British Isles and Aleutian Islands show an increase in some models [58, 65]. Colle et al. [34] found a 10–40 % increase in more intense (<980 hPa) cyclones (Fig. 3b) and 20–40 % more rapid deepening rates just inland of the US East Coast (Fig. 4). These more localized increases have been attributed to the latent heat release within these mid-latitude storms [66], related to the projected increase in water vapor [26].

## Challenges Using GCMs for Extratropical Cyclones

Model spatial resolution is important for the cyclone predictions. At T95 resolution, Jung et al. [67] showed that a global model can only simulate ~60 % of the observed number of cyclones. Several studies have shown that storm track amplitude is positively correlated with increasing horizontal grid resolution [41, 68, 69]. Colle et al. [34] verified 15 CMIP5 models and showed that 6 of the best 7 models over the US East Coast and western Atlantic were the relatively high resolution GCMs (<1.5° spacing). Besides not resolving the strong baroclinicity and associated circulations with these storms, resolving sea surface temperature (SST) and latent heat impacts can also be important, which is the focus of sections 3.1 and 3.2.

### Sea Surface Temperature

There have not been formal studies verifying the SSTs in the CMIP5 models over the storm track regions. Most previous studies have used sensitivity studies to demonstrate the impact of SST distribution in the Atlantic on coastal cyclones. One approach has been to impose an anomalous SST boundary condition on an atmosphere–ocean general circulation model (AOGCM) and compare the results with those from a “control” integration with climatological SSTs. For example, in the Atlantic, the Atlantic “tripole” SST pattern gives rise to a significant response in the NAO [70] and thus cyclones. Minobe et al. [71] illustrated a linkage between the mean Gulf Stream position and the mean atmospheric structure from the boundary layer upwards into the upper troposphere. Nakamura et al. [72] showed a relationship between the mean oceanic SST and the mean wintertime storm track. Although there is still a debate as to whether the key factor regarding the Gulf Stream’s influence on the storm track is the SST gradient [72] or the moisture associated with the warm SST [73, 74].

A few studies have quantified the impact of SST resolution for mid-latitude cyclones in regional models. Jacobs et al. [75] and Joyce et al. [76] used regional weather prediction and climate models over the western Atlantic to show that shifts of the storm track along the US East Coast is related to changes in the Gulf Stream SST gradient. Jacobs et al. [75] showed that an enhanced low-level frontal boundary along this SST gradient induces greater differential turbulent heat flux and thus greater thermally induced surface convergence, which ultimately favors a stronger storm. Small et al. [77] examined how small-scale ocean fronts versus a globally smoothed SST can impact the storm track in the western Atlantic over a 60-year simulation at 50-km grid spacing. There was a modest ~10 % increase of the N. Atlantic surface storm track when the SST dataset resolved ocean front, suggesting that this impact cannot be ignored.

Woollings et al. [78] also showed that the storm track over the western Atlantic is sensitive to how the Gulf Stream SST gradient is resolved in the model. A coarse resolution SST (100- to 200-km grid spacing) yields more storms closer to the US coast than a high resolution (50–100 km) SST. These studies illustrate that there may be changes in extreme winter weather over the Northeast USA as the SST gradients change, and it is important to use regional models with high resolution to assess cyclone changes along the coast.

### Latent Heating and Moisture

**Fig. 5:**
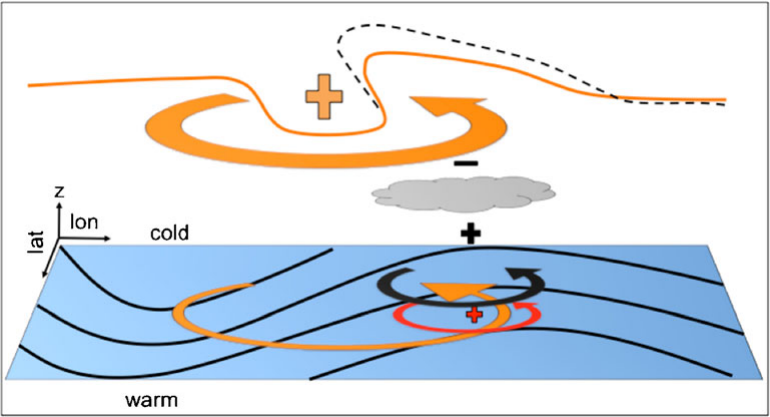
Schematic showing the role of latent heat release in storm intensification in the PV framework. The figure is an update of a schematic for dry circulation, from Hoskins et al. (1985), showing the arrival of an upper-level trough over a low-level baroclinic region (indicated by the *black solid lines*). The *orange* “*plus*” indicates the positive PV anomaly associated with the upper-level trough, and the orange arrows show the circulation associated with this anomaly. The *red* “*plus*” and *arrow* indicate the positive PV and circulation associated with the warm temperature anomaly induced by low-level advection. The *gray cloud* indicates the region of maximum latent heating, and the *black plus and minus* are the PV anomalies associated with the heating gradient. At low-levels, the positive PV anomaly is associated with a stronger cyclonic circulation (*black arrows*), while aloft the negative PV anomaly can be seen as a shift in the location of the tropopause (*black dashed line*)

Latent heating within extratropical cyclones has long been known to influence storm intensity [79]. Due to the regular occurrence of rapid cyclogenesis along the East Coast of North America, the region has been a focus for the study of latent heating in storms, in terms of observations, theory, and numerical modeling [29, 80–86]. The influence of moisture on storm dynamics can be considered in two ways, summarized in Nielsen-Gammon and Keyser [87]: (1) as an external forcing applied to the circulation field, as is done with potential vorticity, or (2) the heating can be incorporated into the vertical advection term in the thermodynamical equation. However, it is the vertical gradient in the heating that changes the circulation (e.g., Martin [88], pp. 290−294). At low levels, the heating gradient translates to a stronger relative vorticity and hence both a faster development of the storm and a stronger maximum intensity [80]; at upper levels, the circulation is adjusted downstream of the jet maximum. A schematic using the potential vorticity (PV) framework to show the influence of latent heating is included here (Fig. 5 [89]). Recent work has improved our understanding of the structure and variability of latent heating within ETs [90–93] and the relative roles of different microphysical properties [94, 95].

The length scales (frontal to mesoscale) and microphysics involved in latent heating make it a difficult mechanism for GCMs to properly capture. In an attempt to test this issue, Bengtsson et al. [51] used a GCM with spectral resolution of T213 (63 km; about half the resolution of CMIP GCMs at the time). For a global warming scenario, Bengtsson et al. [51] found no significant differences in their results compared with earlier coarser resolution experiments, and no significant change in the number of strongest storms (based on storm-averaged 850-hPa relative vorticity). This, they argued, was due to compensation between the strengthening of storms due to more moisture and hence greater latent heating and the damping of storms due to the weakened equator to pole surface temperature gradient. However, the resolution used in Bengtsson et al. [51] is four times coarser than what is typically used in numerical weather models. Related to this, multiple studies have found that the strength of extreme storms, in terms of surface winds and precipitation increases when the horizontal resolution is increased from 100 to 25 km [52, 96, 97].

**Fig. 6:**
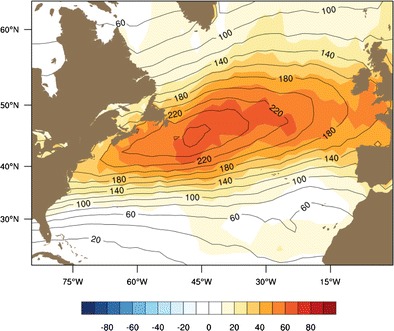
Ten-season variance of highpass-filtered meridional velocity at 300 hPa for 20-km grid spacing (m^2^ s^−2^, *black contours*) and difference (m^2^ s^−2^, *shaded*, 20-km minus 120-km grid spacing). Obtained from Willison et al. [98]. “© American Meteorological Society. Reprinted with permission”

These GCM resolution results motivated experiments that focus on moisture forcing using numerical weather models. In a regional modeling study using a numerical weather model, Willison et al. [98] compared the North Atlantic storm track intensity for simulations using 120 and 20 km. This study found an increase in intensity and intensification in the finer resolution models. For example, Fig. 6 shows that the Eulerian storm track over the North Atlantic has a maximum that is 30 % stronger in the model with 20-km resolution as compared to the model with 120-km resolution. Furthermore, the increase in strength occurs across the entire region of the storm track. Willison et al. [98] show that a significant portion of the increase in storm strength and development speed can be attributed to latent heating. Furthermore, the enhancement of downstream storms in the high-resolution case is related to changes in the upper-level baroclinic wave structure, due to enhancement by latent heating [99].

### Complex Multi-Scale Dynamics

Apart from the challenges involved in resolving sea surface temperature and diabatic heating, many studies have also shown that the dynamics involved in the genesis and evolution of cyclones affecting the Northeast may be complex. Many cyclones have complex circulations in which the initial cyclone occludes and dissipates west of the Appalachians with secondary cyclogenesis over the coastal waters (Miller type B cyclogenesis; [100]). Secondly, upper level waves can approach the Northeast from the northwest along the polar front jet or from the southwest along the subtropical jet [40]. Perturbations along the two jets can interact with each other giving rise to enhanced impacts [101, 102]. Thirdly, given the strong thermal gradient and abundance of moisture in the lower troposphere along the coastal frontal zone, perturbations can grow in the lower troposphere independent of upper level forcing, and interactions between these low level precursors with the approach of upper level waves can lead to enhanced growth [103–105]. In addition, Northeast cyclone development and evolution are influenced by multiple scales [106], including large-scale anomalies such as the PNA and NAO [107], medium-scale downstream development of Rossby waves from eastern Pacific across North America [108], and mesoscale coherent vortices together with latent heating associated with convection [106]. Whether GCMs can accurately simulate the complex interactions between all these processes remains to be assessed.

### Dynamical Downscaling Approaches

The current GCMs are typically run at horizontal grid intervals on the order of 100–200 km, which is too coarse for applications at regional or local-scale regimes at scale of 10–50 km [109]. Therefore, the nested regional climate modeling technique (dynamical downscaling) is often employed to mitigate this problem. He et al. [110] illustrated downscaling approach for a central US cyclone, in which the Weather Research and Forecasting model (WRF) was one-way nested within the Community Earth System Model (CESM). The general purpose of these dynamical downscaling approaches is to add regional detail in response to regional-scale forcing as it interacts with the large-scale atmospheric circulations [111, 112]. However, as noted above, there are large uncertainties in the GCMs, so although the storms look more realistic at higher resolution, we may be downscaling the wrong solutions.

Another form of regional downscaling is called the “pseudo-global-warming” (PGW, e.g., [113–117]), in which perturbations from the GCM future minus the historical are added to the reanalysis-derived initial and boundary conditions used in the control simulations. Marciano et al. [118] used this approach to downscale North Atlantic extratropical cyclones in the CMIP5 members. Rather than running an RCM, Marciano et al. [118] ran case studies of multiple storms, first with current climate conditions and then again with PGW applied. This study also found an increase in intensity, in terms of SLP minimum and 10-m winds and precipitation in the storms in which the synthetic warming was applied. The results suggest a strong influence from additional latent heating within the storms.

There have been few dynamically downscaled model studies of winter extratropical cyclones over the US East Coast and western Atlantic, in which a regional climate model is run within an AOGCM. Long et al. [56] used the Canadian Regional Climate Model (CRCM) with a 30-km resolution embedded in the Canadian Climate Center GCM and showed a potential decrease in extratropical cyclone track density along the East Coast and a slight mean track shift to the northwest during the next 50 years; but it is difficult to gauge the uncertainty in the results since an ensemble of model runs was not performed. Tryhorn and Degaetano [119] used the Hadley Regional Climate Model (HadRM3) with a horizontal resolution of 50 km in conjunction with the Hadley Center GCM HADCM3 to develop projections of heavy precipitation over the Northeast USA.

**Fig. 7:**
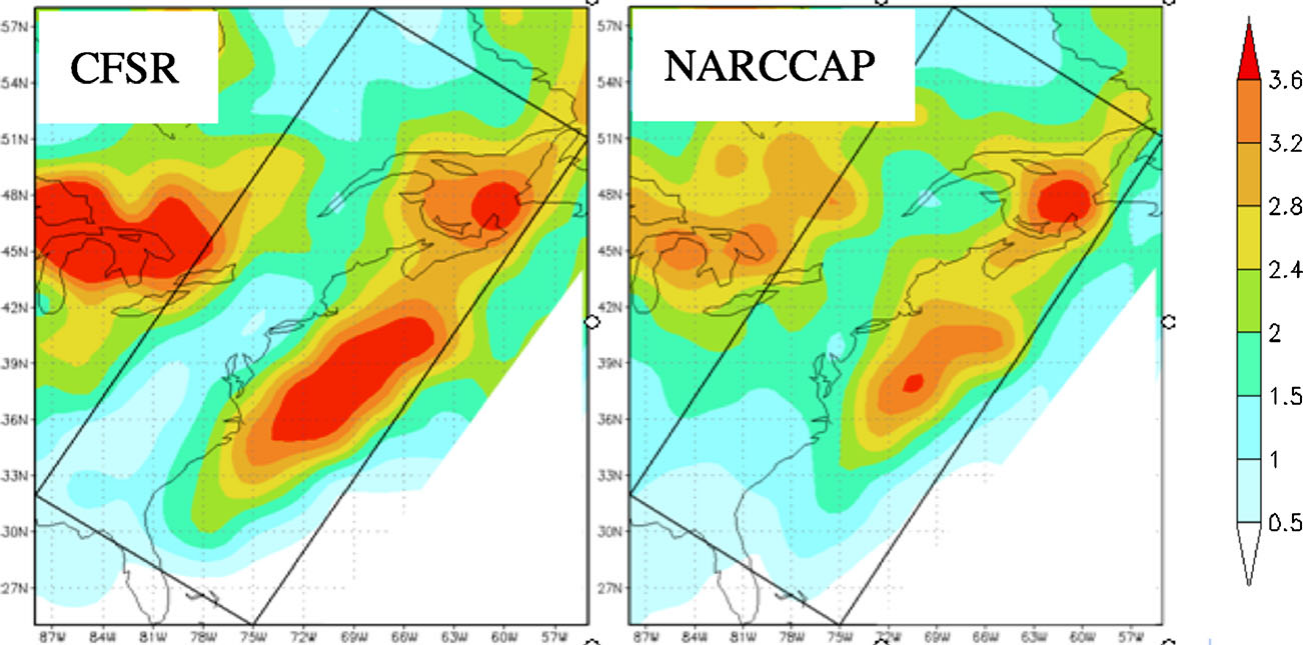
Cyclone spatial density showing number of cyclones per winter (DJF) per 2.5 × 2.5° for the CFSR and NARCCAP mean between 1979 and 1998

A downscaled set of regional runs can have their own set of biases given the physics that are used. This is well known in weather prediction, since a multi-model ensemble of high-resolution models can have fairly large biases [120]; therefore, some sort of bias correction and calibration is often established to improve the predictions (e.g., [121]). In order to illustrate, one downscaled approach has been using NARCCAP (The North American Regional Climate Change Assessment Program: [122, 123]) for 1979–1999. Six RCMs (CRCM, HRM3, MM5I, RCM3, and WRFG) were driven by NCEP Reanalysis and four GCMs (GFDL, CGCM3, HADCM3, and CCSM). The various NARCCAP RCMs use different model map projections and have different domains, but all are run at 50-km grid spacing, while the CFSR uses a 0.5° latitude–longitude grid. Thus, the NARCCAP RCM members and CFSR were interpolated to a common latitude–longitude domain (0.5° × 0.5°). Figure 7 shows the cyclone densities for DJF averaged from six members that were forced by the NCEP reanalysis on the boundary. The CSFR grid was interpolated to the same NARCCAP grid, and a regional version of the Hodges cyclone tracker was used. The NARCCAP underpredicted the cyclone densities by 5–10 % over the western Atlantic, which may be partly due to the close proximity of the downstream boundary over the Atlantic.

**Fig. 8:**
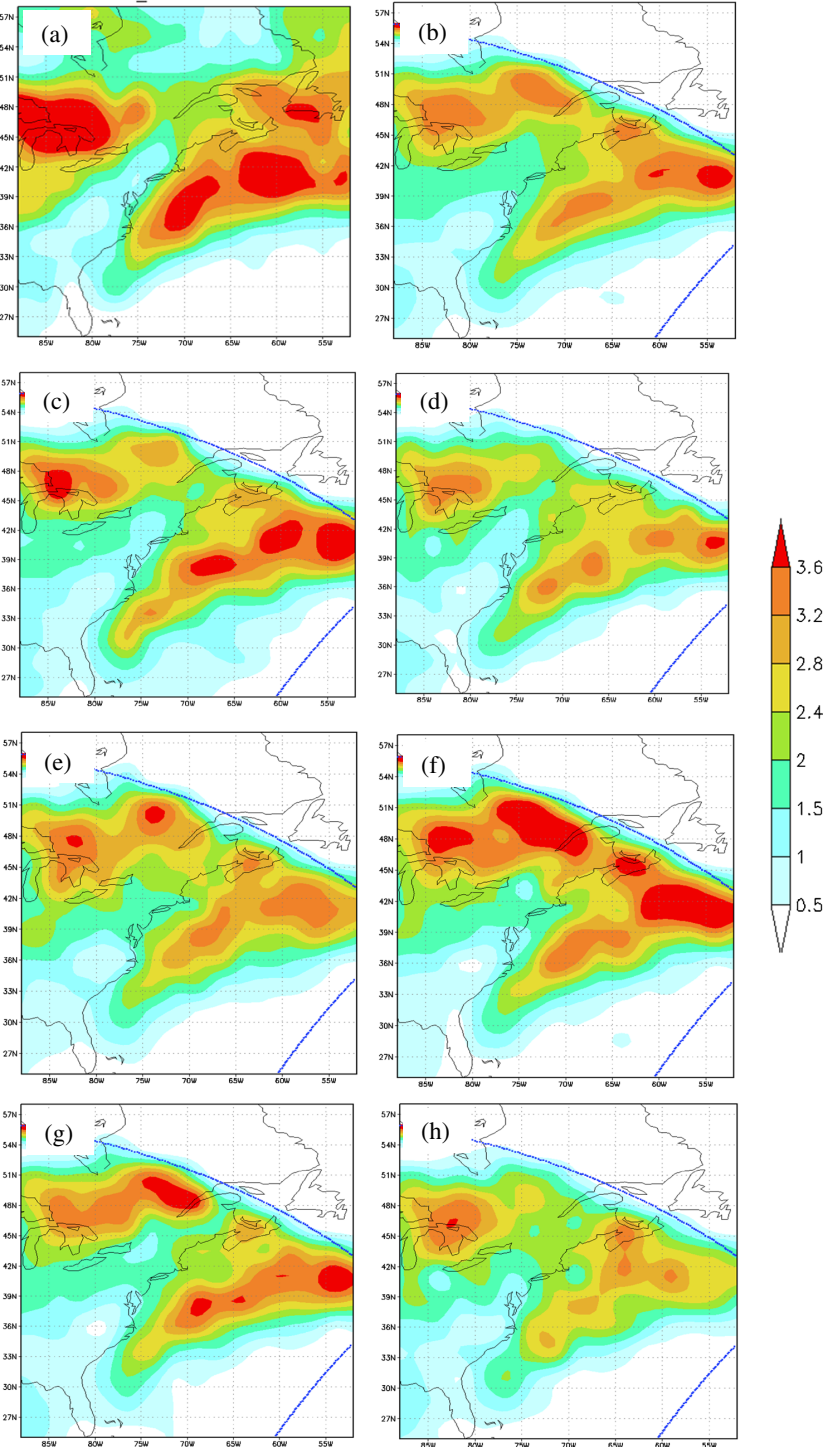
**a** Cyclone density for the CFSR analysis showing the number of cyclones per cool season (DJF) per 2.5 × 2.5° for 1985–2004. **b** Same as (*a*) except for the 8-member WRF member. **c**–**h** Same as (*b*) except for select WRF members

There is also physics uncertainty using just one regional model. For example, an 8-member ensemble of historical simulations were performed at Stony Brook University with version 3.5 of the WRF model, forced by global Reanalysis 2 data (2.5 × 2.5 resolution) for initial and boundary conditions, SST, and snow cover. To develop the WRF ensemble, physics packages within the model were varied, including boundary layer, cumulus, and microphysics. Comparisons were made between surface cyclone tracks from the 8-member ensemble and the Climate Forecast Systems Reanalysis (CFSR) dataset from 1985 to 2004. Figure 8 illustrates the track density of the surface cyclones over the DJF cool season for the 20-year period of interest. Overall, the individual ensemble members capture the coastal cyclone track off the eastern US seaboard somewhat realistically, though there is variability in the number of cyclones, the orientation of the storm track including the proximity of the cyclone tracks from the coastline, as well as the location of the coastal cyclone development region (i.e., entrance to the storm track). This illustrates that there is relatively large sensitivity to downscaled cyclone predictions to the WRF parameterized physics.

## Extratropical Cyclone Impacts

### Heavy Precipitation

Heavy precipitation from extratropical cyclones can have tremendous societal impacts along the Eastern USA, ranging from inland flooding associated with transitional hurricanes to extratropical storms [17, 124] to heavy snow [16]. Pfahl and Wernli [1] showed that as much as 80 % of extreme precipitation events in the ERA-Interim, defined as the 99th percentile of 6-hourly precipitation, is directly related to cyclones over some regions (e.g., Newfoundland, Japan, South China Sea, Mediterranean). Given the projected increase in lower tropospheric moisture [125–127], the number of extreme precipitation events (upper 0.1–10 %) across the globe is anticipated to rise as much as 6–40 % at the expense of lighter events. GCMs may be underestimating this increase [128].

The frequency and annual sum of heavy precipitation over the Northeast USA has been increasing during the last century [129–134]. Wake and Markam [129] showed that there has been 2 % increase in the number of events producing greater than ~50 mm over a 48-h period. Kunkel et al. [133] found that the Northeast has been becoming significantly wetter from 1957 to 2010, and this positive trend has been noted in both observations and GCM ensembles during the winter months [135]. Kunkel et al. [2] showed that extreme precipitation events over the Northeast associated with extratropical cyclones have increased over the past century. Using a select number of CMIP5 models, Maloney et al. [44] showed that the heaviest precipitation events over the Northeast USA may increase by as much as 400 % for the heaviest precipitation events (>25 mm day), albeit the sample size was small (5–25 events over 30 years).

Future variations in regional precipitation during the cool season are a large function of the mid-latitude cyclone track, frequency, and intensity. Lombardo et al. [136] showed that despite a decrease in the number of cyclone days along the US East Coast by the later twenty-first century, there was a 10–15 % increase in the amount of precipitation. This was attributed to a shift towards more frequent extreme precipitation events. However, there has not been work looking at how changes in the size or speed of these storms may impact these precipitation extremes.

### Storm Surge

Nor’easters are responsible for much of the coastal flooding and beach erosion along the US East Coast. Hurricanes typically have stronger wind speeds than nor’easters, but the winds from a nor’easter can last over a few tidal cycles at a particular location and cause significant damage [13, 18, 137]. Storm surge is dependent on a number of factors, such as storm intensity (wind speed), duration, and fetch relative to the coast. Colle et al. [18] showed that NYC moderate storm surge events tend to have a cyclone track along the coast, but there is a fair degree of scatter given that other tracks can still favor a large surge if the onshore (easterly) winds persist for several hours for a large-region offshore. Bernhardt and DeGaetano [138] show that surge caused by extratropical cyclones is more likely to occur when the NAO is in its negative phase and the ENSO is in its positive phase. This, they argue, is because positive ENSO leads to more storms forming near Florida while the negative phase of the NAO is associated with slower moving storms (presumably due to more blocking, though Bernhardt and DeGaetano [138] do not look explicitly at blocking events). Thus, a positive ENSO phase allows for more storms moving up the coast, with easterlies ahead of the warm front pushing water towards the shore. The upper-level jet is weaker during a negative NAO [139], thus giving rise to a slower storm, which can contribute pushing more water toward the shore.

Grinsted et al. [140] noted an increase in coastal flooding during the past century along the US East Coast, while Talke et al. [141] showed an increased coastal flooding for NYC area during the past 100 years, with a large contribution from rising sea level. Currently, there is low confidence in how water levels and waves will regionally change around the globe into the twenty-first century [142]. Recent studies have investigated how storm surges, storm tides, and waves have changed over the twentieth century with the greatest focus on the European coastline/North Sea region using either numerical hydrodynamical models (NHM) [143, 144] or canonical correlation analysis [145, 146].

The problem of quantifying changes to coastal flooding events requires adequate model resolution, but most GCMs underpredict the magnitude of East Coast storms [34]. While dynamical downscaling is one viable method for long-term predictions of storm surge, it is computationally expensive and requires complex methodology to run long integrations utilizing GCM’s with varying grid resolutions. The complex bathymetry and coastal geometry of the Mid-Atlantic Bight also hinder attempts to simulate storm surge with dynamical models for long periods of time as well [8].

**Fig. 9:**
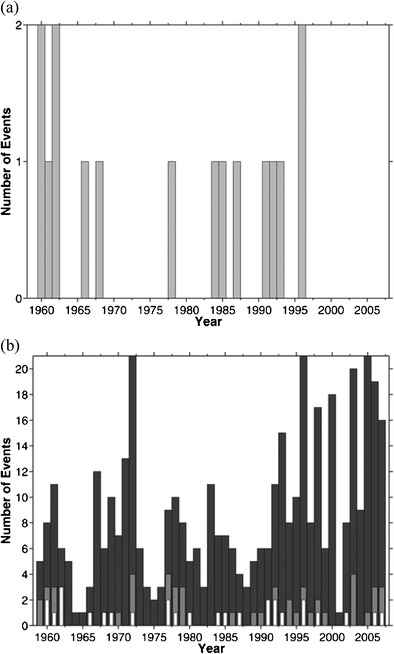
The annual number of observed (storm tide) moderate flooding events at the Battery **a** before and **b** after adding rising sea level from 1959 to 2007, with a 12.5-cm rise (*white bars*), 25-cm rise (*gray bars*), and 50-cm rise (*black bars*). A moderate flood (coastal flood warning for the National Weather Service at the Battery, NYC) occurs when the water level exceeds 2.44 m above MLLW. Obtained from Colle et al. [10]. “© American Meteorological Society. Reprinted with permission”

As a result, there have been no formal studies investigating the future trends of storm surge along the US East Coast for extratropical storms. Meanwhile, one approach is to look at the past trends and consider how that may change for rising sea level [18, 141]. Colle et al. [18] took the number of moderate flooding events at the Battery, NYC (>1.0-m surge) from the mid-1950s to the late 2000s and added sea level rise to determine how the flooding events may change in the future (Fig. 9). There was little change for relatively small sea level rise (0.25 m), with 0–4 events per year. However, for 0.50-m rise, which is a conservative estimate for NYC by late twenty-first century [142, 147], nearly half of the years have 10–20 flooding events per year. Although the future trend of storm frequency and intensity is important, clearly sea level is a major player, such that no change in storm frequency or intensity will result in more frequent flooding around NYC (and many other East Coast locations) in the future.

## Future Directions

As discussed above, climate models still struggle with producing aspects of extratropical cyclones. To address these issues, we first need to properly identify the processes in which the errors occur. A number of process-oriented metric and diagnostic approaches have been applied to understand climate model simulations (e.g., [148]). One climate-model evaluation approach has been using a numerical weather prediction (NWP) approach, by starting the models with realistic initial conditions and quantifying the difference with observations [149–151]. Many recent studies showed how one can use the NWP approach for understanding climate model errors and parameterization improvements (e.g., [151–154]). Two recent examples of more complete diagnostics of CMIP5 model simulations include the Madden Julian Oscillation (MJO;[155]) and East Pacific warm pool variability [156]. There have been no formal attempts completing diagnostics in CMIP5 models for extratropical cyclones. For example, one approach might be a cyclone relative approach, which has been used to understand cyclone cloud structure associated with different precipitation, moisture flux, and temperature gradients [157, 158]. The size and speed of extratropical cyclones have not been formally verified in these models, or how these cyclone properties may change in the future. The surface fluxes and temperature gradients associated with these cyclones depend on the accuracy of the SSTs in the models, but there has not been much verification of the western Atlantic SSTs in the CMIP5 models. Another open question regarding SST forcing of the storms is the relative impacts of mesoscale variability within the Gulf Stream, especially as the ocean components of coupled models move to eddy-permitting grid spacing.

It is clear that improved estimates of regional projections require an ensemble framework. However, the GCMs have varying skill, and it is often not computationally feasible to run tens of members regionally at high resolution. This poses the question of how to select a smaller set of GCM members. McSweeney et al. [159] developed metrics and criteria for model performance, with models removed if their predictions are implausible, having significant errors, and systematic biases. For example, Colle et al. [34] selected CMIP5 models based on their performance of cyclone track density and intensity.

**Fig. 10:**
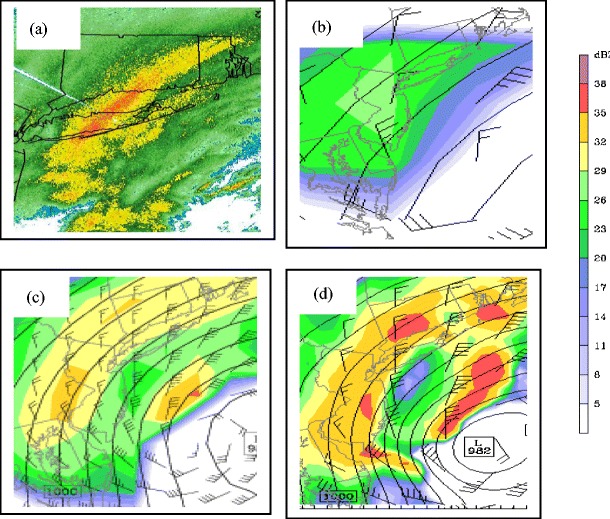
**a** Composite reflectivity (*shaded in dBZ*) around Long Island, NY at 0500 UTC 20 December 2009. **b** Simulated reflectivity (dBZ) from a small portion of the 180-km WRF domain at hour 17 (0500 UTC 20 December). **c**, **d** Same as (*b*) except for a portion of the 60- and 20-km WRF nest

There is a need for more regional climate modeling over the Northeast USA that resolves important mesoscale weather phenomena. Figure 10 shows the importance grid resolution in predicting high-impact weather, such as heavy snow bands over the Northeast USA. On 19–20 December 2009, 40–50 cm of snow fell across Long Island and parts of southern New England. The case was simulated using the Weather Research and Forecasting (WRF) model using a similar setup [16]. In this Novak et al. [16] study, the model grid spacing was 4–12 km; however, Fig. 10 illustrates that the WRF run at 20-km grid spacing over the Eastern USA can realistically simulate these larger bands (Fig. 10a, d). In contrast, the WRF run at 180-km grid spacing (Fig. 10b), which is a similar resolution to many GCMs, cannot simulate the heavy snow and the cyclone is 10–20 hPa underforecast for this event. The 60-km WRF also underestimates the intensity of the snow band (Fig. 10c). These WRF results underscore the importance of using higher-resolution regional climate models in order to answer questions regarding how extreme the weather may change over the Northeast USA.

**Fig. 11:**
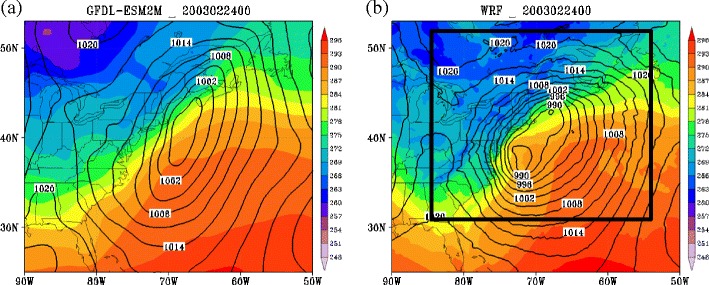
Surface map showing sea level pressure (every 3 hPa) and surface temperature (*shaded every 2 K*) for the **a** GFDL-ESM2M and the **b** 20-km WRF forced using the GFDL-ESM2M at 0000 UTC 24 February 2003 (from historical GFDL run)

**Fig. 12:**
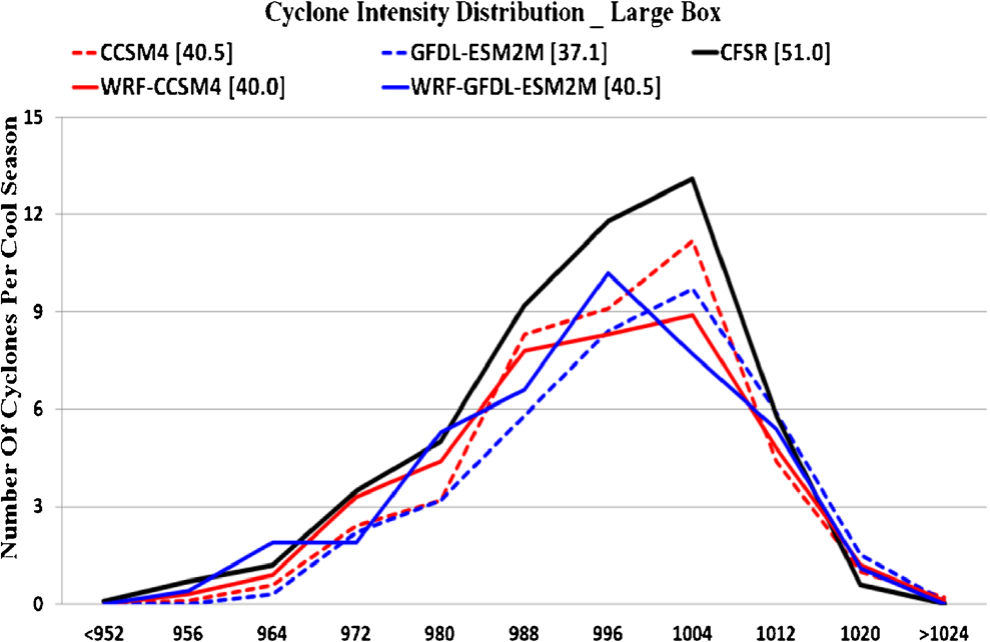
The distribution of cyclone central pressures (in hPa) from January–March 1995–2004 for the GFDL-ESM2M, CCSM4, WRF-CCSM4, WRF-GFDL-ESM2M, and the CFSR for a region covering the Eastern USA and western Atlantic (box in Fig. 11b). The average number of cyclones in the box per cool season is shown with the *number in brackets* in the legend next to each model. The grid resolutions for each model are given in the text

Figure 11 illustrates the stronger storm intensity as a result of using a WRF at 20-km grid spacing nested within a GFDL GCM as compared to the two degree resolution GFDL GCM. As a result, when run over 10 January–March periods (1995–2004), there is a shift in the intensity distribution to more intense storms from the GFDL and CCSM4 models to the 20-km WRF using these models as boundary conditions (Fig. 12). The number of strong cyclones for the 20-km WRF is closer to the CFSR for this period than the GFDL and CCSM4 for the historical period. However, since WRF still uses the GCMs for boundary conditions, the total number of cyclones is close to the GCMs and underpredicted compared to the CFSR. The upper-level disturbances (potential vorticity maximum aloft) responsible for many of the WRF cyclogenesis events enter through the GCM boundary, so if the GCM underpredicts the number of disturbances and associated cyclones, WRF will likely underpredict as well. The impact of boundary conditions will also limit the regional model in terms of cyclone frequency changes relative to the GCMs. As a result, attempts should be made to run a hemispheric high-resolution model (e.g., WRF), to allow more diverse solutions. However, this comes with its own set of challenges, in terms of WRF properly simulating the tropics, arctic, and associated teleconnections. Also, Small et al. [77] illustrated that the small-scale SST gradients can also be important for the western Atlantic storm track; therefore, future downscaled runs need to explore the impacts of SST changes in ocean fronts and eddies.

Section 2.3 summarized results from analyses of the changes in storm track position and strength in global warming projections; however, there are far less diagnostics completed for the reasons for these future changes, which need to separate various physical processes. The low-level temperature gradients are expected to decrease, while the upper-level gradients may be increasing. Meanwhile, in the subtropics, there has been increased upper tropospheric warming and increased stability, which also favors less cyclone activity. As moisture increases in a warmer world, the latent heating will likely increase, and this may offset some of the reduction of the low-level baroclinicity for these storms. These issues regarding changes in baroclinicity are made more difficult along the East Coast because changes in atmospheric stability in the region depend on the land–sea temperature contrast. Moreover, slight shifts in either the polar or subtropical jet may give rise to changes in the complex interactions between perturbations along these jets and the coastal low-level baroclinicity. With global warming, to understand changes in the storm activity, we must understand differences in the response of the land and ocean surface to the warming, as well as the dynamical changes in the ocean circulation.

**Fig. 13:**
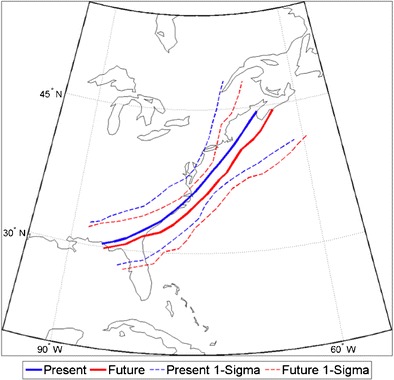
Tracks of the mean present day (*solid blue*) and future (*solid red*) surface cyclones for simulation hours 18–63. The one standard deviation (e.g., 1-sigma) cone for the average present day and average future track is shown by *blue dashed* and *red dashed lines*, respectively. Obtained from Marciano et al. [118]. “© American Meteorological Society. Reprinted with permission”

Marciano et al. [118] used a pseudo-global warming approach in an attempt to capture both the land–sea contrast and the local increase in moisture. However, by imposing the projected mean state changes onto the initial conditions of a present day storm, they may have introduced a bias in the storm development. They found that under pseudo-warming conditions, the storms’ tracks were east of present day tracks (Fig. 13), and this change is consistent with an intensification of the upstream, upper-level jet in their model. Marciano et al. [118] attribute the change in path to the moist physics within the storms (a self-development mechanism); however, it may be the changes in the upper-level jet that caused the change in the path. This projected change in the path of the tracks is interesting because the Marciano et al.’s [118] eastward track shift differs from that found in the CMIP5 ensemble by Colle et al. [34]. Regardless, the disagreement highlights the need for more analysis of the changes in cyclone path in response to changes in the mean state and the local heating.

When considering the change in moisture, one must also consider the global role of extratropical cyclones in poleward moisture transport [25]. As highlighted in the discussion above, increased moisture content allows for more condensation within the storm, which has been shown to strengthen individual storms (e.g., [160]). However, an increase in moisture content within poleward-moving circulations also increases the net poleward energy transport, meaning that less or weaker storms are needed to remove the equator to pole temperature gradient [161, 162]. Thus, two issues need to be addressed in future research: cloud and precipitation processes in GCMs need to be improved and lateral boundary conditions in regional climate models need to include the global energy impacts of well-resolved moisture transport (perhaps through improved two-way nesting).

As noted in the previous section, downscaling using dynamical models is expensive, especially if an ensemble approach is implemented. This should be attempted, but another approach is to use a statistical downscaling technique for some phenomena. For example, one could use bias-corrected GCM output around an area of interest and develop statistical approaches relating storm surge at a point to the winds and pressures in that region. Roberts et al. [163] developed a multi-linear regression approach to predict storm surge at the Battery, NYC. For a specified region to the east and southeast of the Battery, prolonged surface stress and sea level pressure minimum are used as predictors. The model is shown to have nearly the same accuracy as an operational dynamical surge model. Therefore, this statistical model can be used with CMIP5 models to look at future trends of surge for NYC.

There is also a need for more work on severe winds associated with extratropical cyclones. Pryor et al. [164] found spatial coherence over distances of up to 1000 km in strong surface wind events, which, as they point out, implies synoptic systems create the wind events. Booth et al. [165] show that there is a preferred path for storms associated with severe surface winds: approaching the Northeast USA from the west/southwest. However, links between future projections of storms and the wind events have not been made.

The majority of the studies reviewed here highlight the frequency and intensity changes of extratropical storms. However, some of the more devastating storms, such as hurricane Sandy, were transitioning storms from a hurricane to an extratropical cyclone as they approached the coast. These storms, often referred to as tropical–extratropical transitions, or ET storms ([166, 167] for review), possess hybrid characteristics of tropical and extratropical cyclones, such as a warm core, but the presence of low-level temperature gradients. There have been future climatologies of tropical cyclones along the East Coast for storm surge [168], and extratropical cyclone climatologies [34], but little analysis of these hybrid storms. Thus, an effort is needed to better understand the future trends of these hybrid storms along the US East Coast (e.g., Sandy 2012).
